# A 22-year-old woman with fever, shortness of breath and chest pain

**DOI:** 10.4103/1817-1737.53345

**Published:** 2009

**Authors:** G. L. Meena, Gaurav Jindal, Rajul Rastogi, Kamal Jindal

**Affiliations:** *Department of Radiodiagnosis, Sardar Patel Medical College, Bikaner, Rajasthan, India*

A 22-year-old woman was admitted to our institute with complaints of fever for the past 5 days; cough, chest pain and progressively aggravating breathlessness for the past 2 days. There was no significant past history. On examination she appeared moderately ill; temperature was 100°F, heart rate was 100/ min and respiratory rate was 22/min. Chest examination revealed decreased breath sounds bilaterally.

## Clinical Questions

1. What are the radiological findings?
2. What is the likely diagnosis?

## Answers

**Figure 1:**
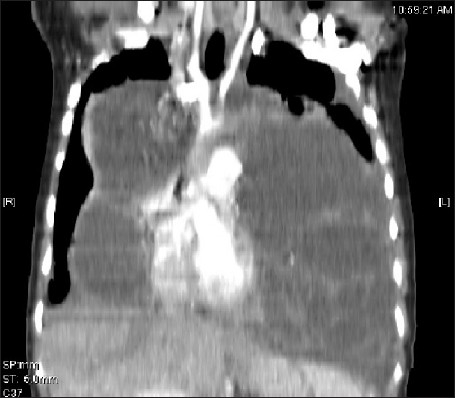
CT scannogram

**Figure 2:**
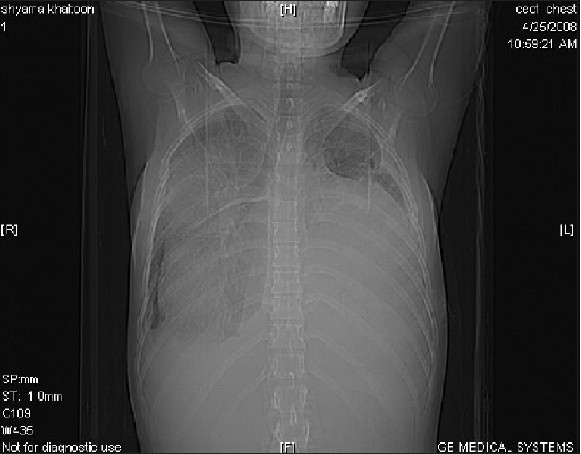
Transaxial CT image

**Figure 3:**
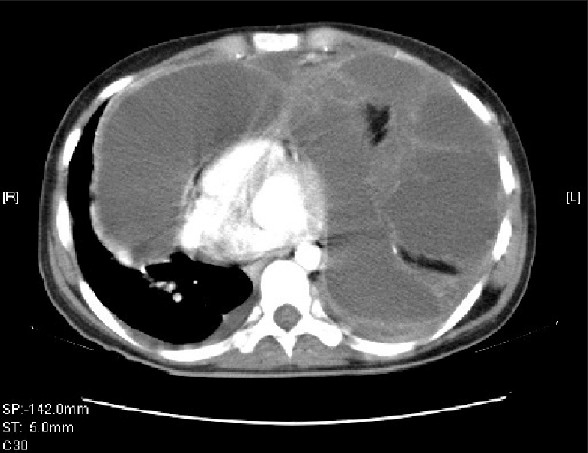
Re-formatted coronal CT image

1. CT scannogram [Figure 1] shows a homogenous opacity causing widening of the mediastinum and obscuring a large part of bilateral lung fields. Transaxial CT images reveal a large well-defined cystic mass measuring 21 × 17 × 16cm with a volume of 1904 cc in the anterior mediastinum, compressing the trachea, great vessels and cardia posteriorly and extending to both sides of the midline, causing compressive atelectasis of large parts of adjoining lung parenchyma. Multiple enhancing septations, areas of fat attenuation and calcification are seen within the mass [Figure 2]. Coronal re-formatted image displays the huge mass extending into both hemithoraces [Figure 3]. There is no evidence of invasion of the airway or vessels.
  Preoperative diagnostic aspiration was attempted, which revealed turbid fluid that showed numerous neutrophils on cytology.
2. CT scan findings of a well-defined multilocular cystic mass with areas of soft tissue, fluid and fat attenuation and calcification, and the presence of neutrophils on cytology suggest a diagnosis of an infected mature teratoma.
  The patient subsequently underwent a thoracotomy. A large complex well-defined mediastinal mass was found, which showed multiple areas of adhesion to the surrounding lung and pericardium. The adhesions were separated and the mass was completely excised. She recovered well from the operation. Postoperative chest X-ray (CXR) showed well-aerated bilateral lung fields. Preoperative lung atelectasis had completely resolved. Histopathological analysis of the resected specimen confirmed the diagnosis of a benign mature teratoma. She has been well for 4 months postoperatively.

## Discussion

Germ cell tumors are classified into benign forms, which include mature teratomas; and malignant forms such as seminomas, malignant teratomas, embryonal carcinomas, choriocarcinomas and endodermal sinus tumors.[1] Benign teratomas represent 3% to 12% of mediastinal tumors.[2] These tumors arise from primordial germ cells that fail to migrate to the gonads during embryonic development.[1] The first example of a mediastinal teratoma was described by J. A. Gordon in 1823. They are most commonly seen between the 2^nd^ and 4^th^ decades of life, with an equal distribution with regard to sex.[3]

Teratomas are slow-growing tumors and are mostly discovered incidentally on chest radiographs performed for other reasons. Symptoms arise from compression of adjacent structures, mainly the cardia, great vessels and airways; and from compressive atelectasis of surrounding lung parenchyma. Secondary infection or hemorrhage into the mass or malignant degeneration is known to cause a rapid increase in size of the mass, making it symptomatic.[4] Trichoptysis is considered to be a pathognomonic sign, which results if a communication develops between the mass and the tracheobronchial tree.

Imaging is crucial for arriving at the correct diagnosis. Chest radiographs reveal a well-defined round or oval mass in the anterior mediastinum extending to one side of the midline. Areas of calcification, well-formed teeth or bone may be visualized. Chest CT scan establishes the diagnosis. It readily detects fluid, fat, calcification and soft tissue densities. Mature teratoma appears as a well-defined multilocular (less commonly, unilocular) cystic mass with areas of soft tissue, fluid and fat attenuation and calcification or a variable combination of the four. Calcification may be focal or rim like; well-formed teeth or bone may also be seen. CT accurately delineates the extent of the mass and also displays its relationship to the adjoining mediastinal structures. Findings on chest MRI include a heterogeneous signal-intensity mass containing a variable combination of fluid, fat, soft tissue and calcium.[3,5,6] Ultrasound has shown promising results in the evaluation of mediastinal masses, especially in the upper mediastinum. It displays the cystic nature of the mass, internal septations, wall thickness, vascularity and appearance of internal fluid. Mediastinal teratomas show 3 sonographic patterns, which include complex mass of heterogeneous echogenicity, homogeneous high echogenicity within a solid mass and floating spherules within a cystic mass.[7,8]

Benign teratomas can be differentiated from malignant germ cell tumors by the presence of greater amount of soft tissue, ill-defined margins and invasive features in the latter.

The combination of fluid, fat, calcification and soft tissue densities is highly specific of a teratoma and is helpful in distinguishing teratoma from other anterior mediastinal masses such as thymoma or lymphoma.[6]

Teratomas can get infected from contiguous foci in the lung parenchyma or via the bloodstream. With onset of infection, there is a rapid influx of fluid and sudden increase in size of the mass, turning it symptomatic as seen in our case.

Mature teratomas are considered chemo- and radio-resistant, and surgical excision is the treatment of choice. Complete excision is considered curative.[3]

Mediastinal teratomas are usually asymptomatic and discovered incidentally on chest radiographs. This is a rare presentation of a benign teratoma presenting as a huge mediastinal mass with characteristic radiological features, extending into bilateral hemithoraces and coming to clinical attention with acute-onset respiratory discomfort secondary to infection within the mass. The diagnosis was accurately established preoperatively on the basis of characteristic CT scan findings. Such a large mediastinal teratoma has only rarely been reported previously.
